# Vibrational contribution to the sub-terahertz dielectric response of kinesin and its hydration shell

**DOI:** 10.1038/s41598-026-40625-0

**Published:** 2026-03-01

**Authors:** Saurabh K. Pandey, Michal Cifra

**Affiliations:** Institute of Photonics and Electronics of the Czech Academy of Sciences, Prague, Czechia

**Keywords:** Biophysics, Physics

## Abstract

The ability of proteins to change conformation underlies both their biological function and their use in bio-nanoelectromechanical systems as molecular machines and transducers. These conformational transitions are suggested to be facilitated by global physical vibrational modes in the sub-THz frequency range. However, direct experimental detection of these modes is difficult to achieve due to their weak spectroscopic signatures and strong water background. Thus, computational approaches fill knowledge gaps, help steer and interpret experiments. In this study, we used molecular dynamics simulations combined with normal mode analysis to explore the vibrational modes of an all-atom model of the globular motor domain of protein kinesin. We explored the coupling of these modes, via the corresponding modal dipole variation, to the electromagnetic field and predicted the resulting dielectric properties and absorption spectra in absolute units. We found that the inclusion of a water layer in the system leads to a blue-shift and reduced amplitude in the absorption spectra in the low-frequency (0-400 GHz) region. Further decomposition of absorption spectra into protein and water components showed non-additive behavior, arising from partially antiparallel dipole variation vectors of the protein and water fractions, which reduced the overall absorption. Comparison showed that the tubulin heterodimer has stronger absolute sub-THz absorption, consistent with its larger molecular weight and, hence, higher number of atoms and degrees of freedom than kinesin. Together, these results provide a mechanistic understanding of hydration effects on vibrational modes and dielectric properties of proteins. In a greater context, the results have implications for methods in bio-nanoelectromechanical systems for protein dynamics and conformation sensing and in biomedicine and bioelectromagnetics for electromagnetic field-mediated functional modification of proteins.

## Introduction

Proteins represent the most abundant and versatile biological macromolecular class performing a wide range of essential functions, ranging from enzymatic catalysis and signal transduction to molecular transport and structural support^1–6^. A protein’s ability to switch between conformational states underlies many of these functions. Protein normal mode analysis (NMA) plays an important role in elucidating conformational transitions between distinct states. First, NMA and related methods are well-established as modelling approaches for mapping reaction coordinates and the energy landscapes on which these transitions occur^7–12^. Second, several authors have argued that the vibrational modes obtained from NMA and related techniques correspond to physically real degrees of freedom (and are therefore potentially accessible spectroscopically)^13–15^, and that these vibrational modes are functionally relevant for facilitating energy transfer, enzymatic catalysis, regulating allosteric transitions, and enabling large-scale motions and conformational changes^16–19^.

Due to the mechanical properties of proteins, the collective vibrational modes are suggested to span frequencies from gigahertz to several terahertz^16,19–21^. Protein vibrations in this range can be probed experimentally by various spectroscopic methods such as terahertz time-domain spectroscopy^22,23^, neutron scattering^24,25^, Raman and infrared spectroscopy^14,26–29^; however, it remains difficult to identify functionally relevant low-frequency vibrational modes due to their weak spectroscopic signatures compared to background^17,30^. Whether for enhancing enzyme activity, improving antibody or drug design, or advancing other forms of protein engineering, these applications ultimately rely on a deeper understanding of collective motions. Developing such an understanding is therefore essential not only for progress in applied bioengineering but also for elucidating the fundamental biological processes these vibrations underlie.

Water, as the natural solvent for proteins, adds a crucial yet complex dimension to this problem. Water is responsible for proper folding of proteins; hence, it influences the structure and, in turn, the dynamics of a protein molecule^31,32^. The role of water in protein dynamics and vibrational modes has been probed by various spectroscopy methods^22,33,34^. However, the extent to which hydration affects low-frequency vibrational modes remain insufficiently analyzed. The presence of water makes it challenging to capture the low-frequency motions of protein via THz or IR spectra absorption because of the strong water absorption compared to that of protein. One way to solve this problem is to keep just enough water around protein molecules to preserve their native structures but minimize bulk water absorption^19,35^. The water molecules in a system can be divided into bound and bulk (free) water^36,37^. However, there is no consensus on how to define the extent of bound water or how many hydration shells directly affect or contribute to the functional dynamics of proteins. Long-range protein–water interactions up to 20 Å have been suggested to affect protein-water dynamics or absorption by experiments^33^, but it is not clear how water amplifies or cancels absorption. Furthermore, many theoretical spectra are presented in arbitrary units; converting simulations to physically measurable absorption and permittivity (with realistic concentrations and linewidths) is essential for validation, yet not consistently performed.

In this paper, we investigated the normal vibrational modes of a motor domain of a kinesin protein. Kinesin is essential for various cellular processes, from cell division to intracellular transport^38^. In cell division, kinesin generates forces that facilitate spindle pole separation, spindle bipolarity and organization, chromosome positioning, and congression^39,40^. It is also essential for the transport of subcellular cargo, such as vesicles and organelles^39,41^. For instance, in neurons, kinesin moves synaptic vesicles from the cell body to the axon terminals, making sure that neurotransmitters and neuroreceptors are delivered to their intended destinations^42^. For kinesin, conformational transitions appear to be mapped onto collective contributions from multiple vibrational modes rather than being dominated by only the lowest few; several tens of modes may participate in the transition^43^.

**Fig. 1:**
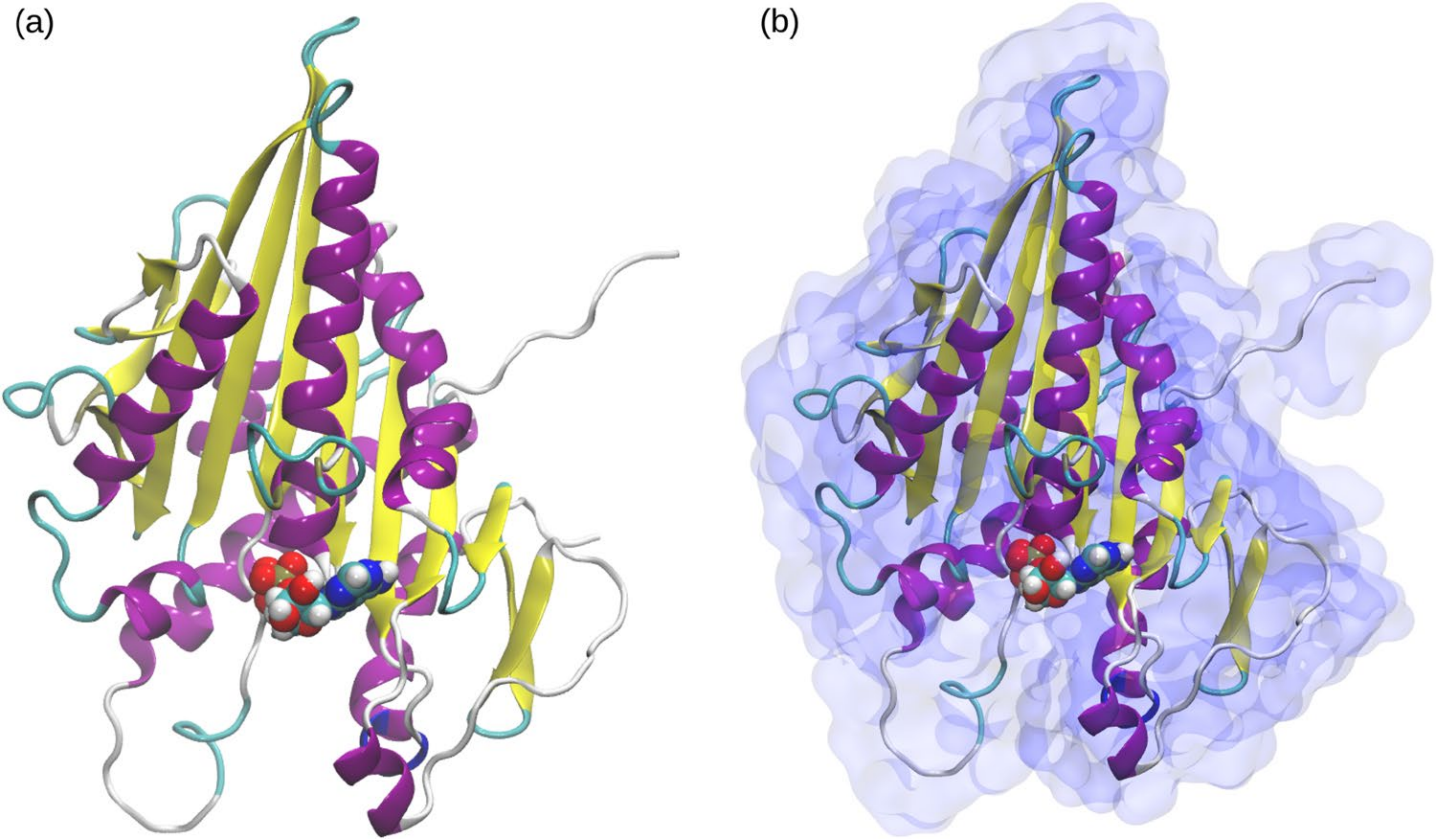
Kinesin motor domain analyzed in modelling: (**a**) kinesin motor domain (shown as cartoon) with adenosine diphosphate (ADP) and magnesium ion ($\hbox {Mg}^{2+}$) (shown as VdW - Van der Waals - spheres), (**b**) Kinesin-ADP-Mg complex with a 3 Å thick water layer. $\alpha$-helices in magenta, $\beta$ strands in yellow.

Theoretical and experimental evidence suggests that the natural frequency of protein vibration modes lies in the sub-THz to THz range^14,16,17,44,45^, depending on the protein’s mechanical properties and the particular vibrational mode^15,19,26,46^. Because proteins contain bound charges, structural oscillations due to particular vibrational mode also modulate their charge distribution, resulting in a dipole moment that varies with the frequency of the vibration. An external oscillating electric field can therefore drive oscillatory variations of the dipole associated with a particular vibrational mode^13,47^. This effect can be used for probing protein dynamics using an external electric field as well as for driving the oscillations to affect the protein dynamics, if the electric field was strong enough.

Here, we combined molecular dynamics simulations with normal mode analysis to investigate the vibrational modes of the kinesin motor domain. Specifically, based on the normal mode analysis (NMA), we (i) computed eigenmodes and their frequencies (ii) associated dipole moment variations (iii) evaluated dielectric properties and absorption spectra (iv) quantified the effects of hydration layers on vibrational response (v) compared kinesin with tubulin to identify structural-dynamical factors affecting sub-THz absorption (vi) predicted effects of protein concentration and damping on the absorption spectra.

## Results and discussion

First, we focused on the kinesin monomer (ADP–Mg bound) without any surrounding water to establish a reference for its intrinsic normal modes (Fig. 1a). Next, to evaluate how hydration influences the eigenmodes and their frequencies, we included a 3 Å water layer around the protein (Fig. 1a). In general, the addition of water significantly increases the computational complexity, requiring more time for energy minimization, Hessian matrix generation, and diagonalization; therefore, including a full water box in NMA calculations was not feasible. However, such extensive solvation is not even required for our comparative purposes, since water molecules located beyond 3 Å are much less coupled to protein motions, as will be discussed later. Fig. 1 shows the kinesin monomer with and without the 3 Å water layer, representative of 92 structures for each type of system, extracted from MD trajectories previously reported^48^.

### Water dynamics and hydration shells

Water molecules close to the protein exhibit dynamical properties distinct from those of water molecules away from protein^36,49^. These water molecules form a hydration shell, also called biological water or bound water, influence the structure, function, flexibility, and dynamics of protein molecules^31,32^. However, it is often difficult to draw a clear boundary between bound and bulk water^50^. Recent experimental and computational studies have shown that the dynamical properties of hydration water around biomolecular surfaces form a continuous spectrum rather than two distinct regimes. The mobility and relaxation dynamics of water molecules are moderately slowed near protein surfaces and gradually recover toward bulk values with increasing distance, exhibiting spatially and temporally structured behavior^51–53^. These findings suggest that instead of defining a sharp virtual boundary or discrete number of water shells, it is more appropriate to consider a graded hydration layer whose properties vary continuously with distance and local environment.

**Fig. 2:**
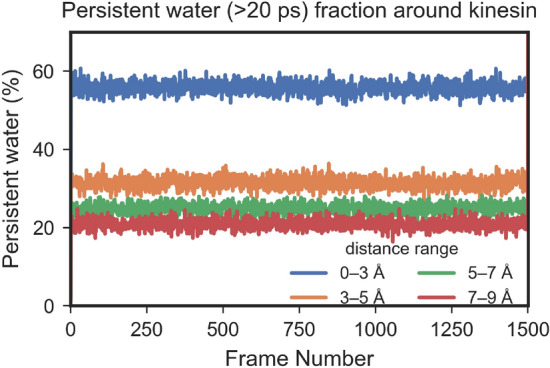
Percentage of water molecules in four water shells (0–3, 3–5, 5–7, 7–9 Å) that remain within the same shell for $\ge$ 20 ps, plotted over the 1500 saved MD frames (△t = 20 ps per frame).

We investigated the dynamics of water molecules in different water shells around protein in 30 ns long MD simulation trajectories. In these trajectories frames were saved at every 20 ps; thus 1500 frames represent a 30 ns (30,000 ps) long simulation. Four water shells around the kinesin protein structure were defined: 0–3, 3–5, 5–7, and 7–9 Å. The number of water molecules that stayed within a particular shell for $\ge 20$ ps was considered persistent water in that shell. Fig. 2 shows four lines that correspond to water shells. More than half of the molecules present in the 0–3 Å water shell at any given time point remain within the shell for at least 20 ps. The persistent water for other shells is much lower than that in the nearest water shell (0–3 Å) to the protein, and it decreases as the shell’s distance increases from kinesin. These data clearly demonstrated the different dynamics of water molecules in different water layers and prompted us to consider water molecules within 3 Å as bound water and include those as a water layer in the normal mode analysis.

**Fig. 3:**
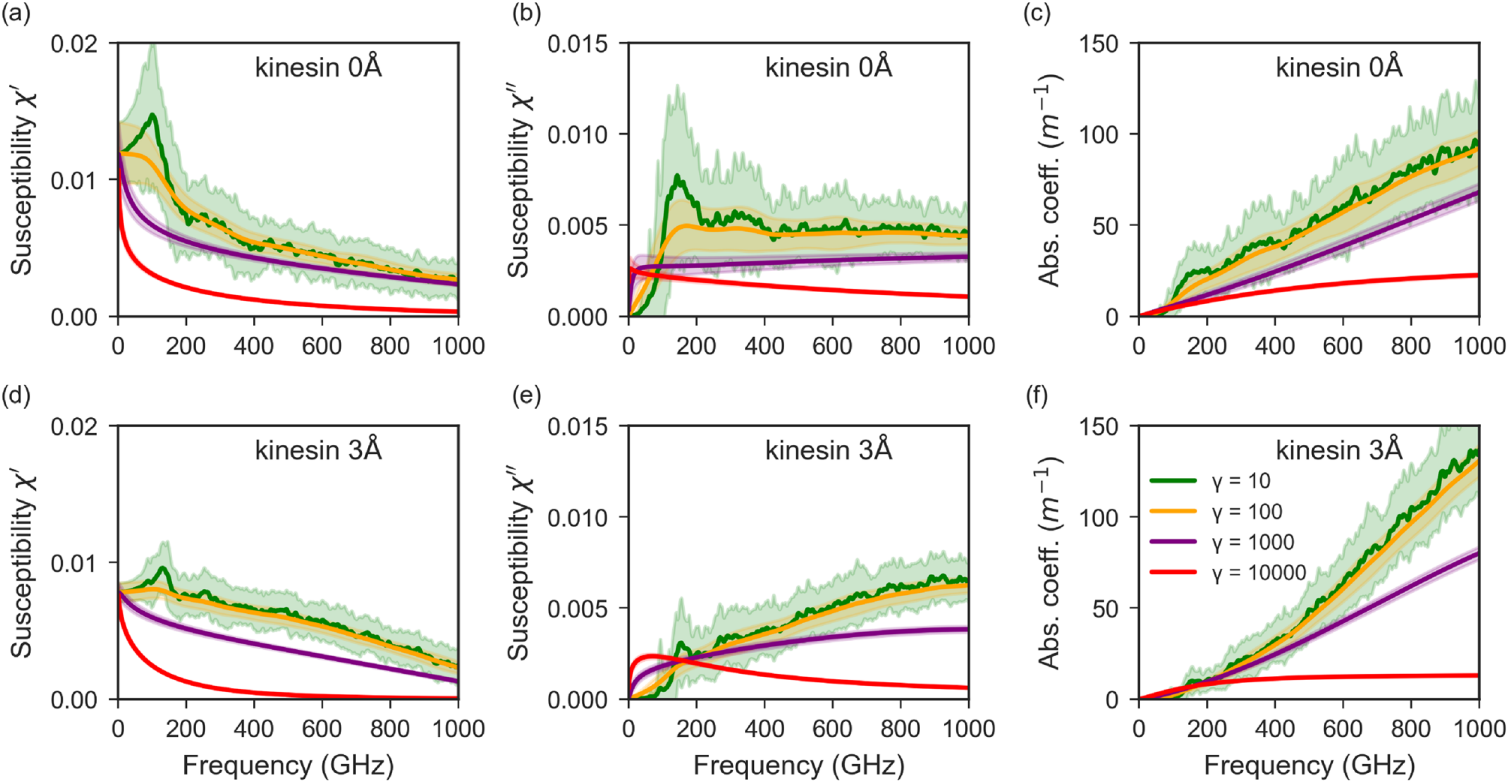
Dielectric spectra due to vibrational motion of kinesin system, obtained from NMA either from dry protein (kinesin 0 Å) or with 3 Å water layer (kinesin 3 Å), as a function of damping (from $\gamma =10~$GHz (green) to $\gamma =10,000~$GHz (red) : (**a**, **d**) real (**b**, **e**) imaginary part of relative susceptibility (**c**, **f**) absorption spectra. The concentration of kinesin is 19 mg/mL (0.5 mM).

### Susceptibility and absorption

The normal modes represent collective motions of the system (protein or protein-water) where each normal mode is associated with a certain shape and frequency of oscillation. A protein structure has a certain spatial arrangement of atoms carrying charge; hence every protein structure or conformation has an associated molecular dipole known as a static molecular dipole. A normal mode represents an oscillatory change of the spatial arrangement of these charge-carrying atoms, which leads to a change in the molecular dipole. This change associated with a normal mode or eigenmode is known as the variation of the dipole moment, hereafter represented as $\rho$. Since each of the vibrational modes has an associated frequency and variation of dipole moment, the system can be considered as an oscillator that can interact with an external electromagnetic field. This makes NMA a useful tool to predict spectroscopic properties of proteins. Using Eq. 2, we calculated the variation of the dipole moment of the first one thousand eigenmodes of kinesin-only and kinesin-water systems. Furthermore, we applied Eqs. 3 and 4 to calculate the imaginary and real parts of susceptibility, respectively. The real part of the susceptibility ($\chi ^{\prime }$) reflects the capacity of the system to store energy under an external electromagnetic field, while the imaginary part ($\chi ^{\prime \prime }$) characterizes energy dissipation and is directly related to absorption. In addition, we analyzed the effect of damping on dielectric properties and absorption spanning regimes from underdamped ($\gamma$ = 10 GHz) to strongly overdamped ($\gamma$ = 10,000 GHz).

The comparison of frequency-dependent dielectric response of two types of systems, kinesin-0 Å (no water) and kinesin-3 Å (with 3 Å water layer), reveals distinct spectral features (Fig. 3). The real part of susceptibility (Fig. 3a) for kinesin-0 Å (no water) system shows a peak in the low-frequency region around 140 GHz. This peak indicates slow collective modes in this low-frequency region suggesting large-scale motions in the system. Corresponding to these motions, dielectric loss appears as a broad peak in the imaginary part (Fig. 3b) in the frequency range from 140–200 GHz. In contrast, in the kinesin-3 Å system $\chi ^{\prime }$ peak at 140 GHz is reduced; however, the higher-frequency modes (frequency range 200–600 GHz) show enhanced susceptibility(Fig. 3d). The imaginary part of susceptibility $\chi ^{\prime \prime }$ for kinesin-3 Å also showed a narrow and lower peak around 140 GHz (Fig. 3e) and lower values in 200–500 GHz range compared to kinesin-0 Å (Fig. 3b). After 500 GHz the $\chi ^{\prime \prime }$ values keep increasing in kinesin-3 Å (Fig. 3e) whereas in kinesin-0 Å, the values do not increase, represented by a flat green line in Fig. 3b.

The absorption spectra of kinesin-0 Å (Fig. 3c) show a small and broad peak around 140 GHz corresponding to the energy loss/dissipation observed as a peak in $\chi ^{\prime \prime }$ (Fig. 3b). Compared to this, kinesin-3 Å shows less absorption in this low-frequency region (Fig. 3f). Instead, there is a blue-shift in the absorption, indicated by higher absorption values after 600 GHz. The observation of a blue-shift of the lowest frequency vibrational mode is consistent with our early observations in our recent paper on tubulin NMA^54^ - the protein system with its hydration shell becomes effectively stiffer. To understand why the values of $\chi '$, $\chi ''$, and the absorption in the low-frequency range (below 400 GHz) are smaller for kinesin–3 Å than for kinesin–0 Å, we examined the magnitude of the underlying dipole variation, $\rho$. Fig. S1-3 shows that $\rho$ values are consistently lower for kinesin–3 Å compared to kinesin–0 Å. According to Eq. S2-1, this difference directly leads to reduced values of $\chi '$, $\chi ''$, and absorption. The physical origin of this reduction within the system will be discussed in the next section.

### Contribution of protein and water in the absorption and dipole direction

**Fig. 4:**
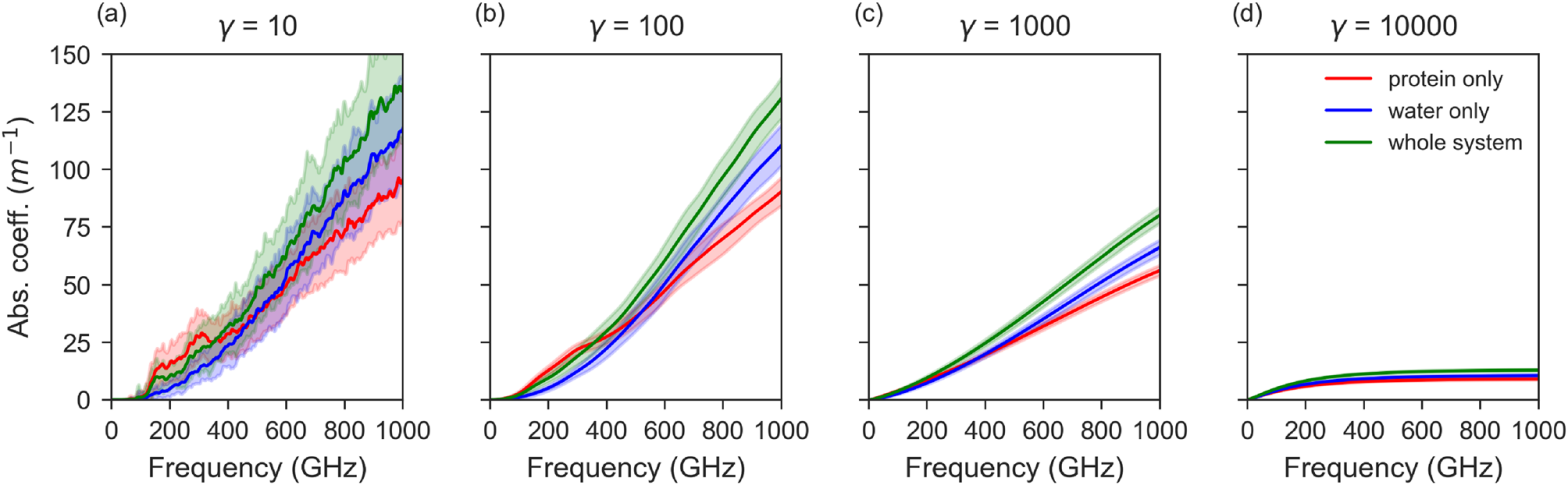
Absorption spectra of kinesin 3 Å system: contribution of protein and water components obtained from normal mode analysis to the absorption spectrum and the effect of damping in kinesin with 3 Å water layer. Panels (**a**–**d**) correspond to different damping constants, $\gamma = 10$, 100, 1000, and 10000 GHz, respectively. The spectra illustrate the effect of increasing damping on the absorption of the protein-only (red), water-only (blue), and whole-system (green) components. The concentration of kinesin is 19 mg/mL = 0.5 mM.

To understand the vibrational contribution of the water shell to absorption and other dielectric properties, we dissected the contribution of protein, water, and whole system (kinesin-3 Å), see Fig. 4. While the source NMA data are common to the whole system, the protein and water parts of the system, for the absorption calculations (Eq. 6) we constructed $\rho$ using only the atoms that belong to the corresponding component. Four panels in the Fig. 4 show absorption spectra in underdamped as well as in overdamped systems. Separating the absorption into contributions from the protein and water components reveals several interesting features in the spectrum. The absorption due to a particular component seems to be frequency dependent. In the frequency range of 150–400 GHz (Fig. 4a), the protein exhibits higher absorption than water. Above this range, the absorption of water increases more rapidly and exceeds that of the protein. In the 150–350 GHz frequency range, the absorption due to the whole system is lower than absorption by protein-only in some parts, see e.g. Fig. 4a. Another noteworthy feature is that at no frequency across the examined spectrum does the absorption of the whole system equal the simple arithmetic sum of the individual contributions from protein and water. Instead, the system behaves in a more complex manner, suggesting the presence of interactions and coupling effects between protein and water that modify the overall absorption response.

**Fig. 5:**
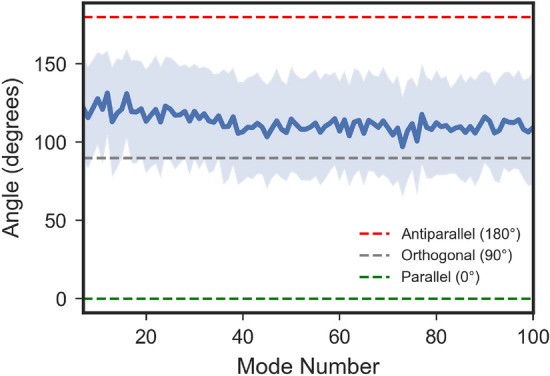
Angle between dipole variation of protein and water in kinesin 3 Å system.

Since the major determinant of the absorption in protein and water components primarily is the variation of dipole moments of each component, we investigated the direction of $\rho _j$ for protein and water for each mode. The angle between $\rho _j^{Protein}$ and $\rho _j^{Water}$ was calculated for each mode and each of the 92 models^54^. The average of this angle over 92 models is shown in the Fig. 5 for the first one hundred modes. The mean values of the angle between $\mathbf {\rho }_j^{Protein}$ and $\mathbf {\rho }_j^{Water}$ are slightly obtuse fluctuating around $120^\circ$. Clustering of angles around $90^\circ$ would have meant that angles between these two dipoles have a random distribution^55^. However, the systematic bias toward angles larger than $90^\circ$ (closer to antiparallel than parallel) suggests a structural-dynamical anticorrelation between protein and water dipole variation vectors. The angles here do not show strong mode-dependent trends, but rather fluctuate moderately within a relatively stable band and a mild decreasing trend towards higher mode numbers. The predominance of angles above $90^\circ$ means that protein and water dipoles tend to orient in a partially opposing fashion rather than cooperating in parallel. This partial cancellation of transition dipoles likely explains why absorption by a whole system is not equal to a sum of absorption by individual protein and water components, see Fig. 4. Our finding of partial cancellation of protein vibrational response by water shell vibrational response is corroborated by molecular dynamics simulations of hydrated protein THz absorption in another work^56^, where protein-water interaction (protein-water dipole cross-correlation) was found to strongly cancel the protein contribution (protein dipole autocorrelation) to overall absorption.

### Absorption spectra of tubulin dimer and kinesin monomer

**Fig. 6:**
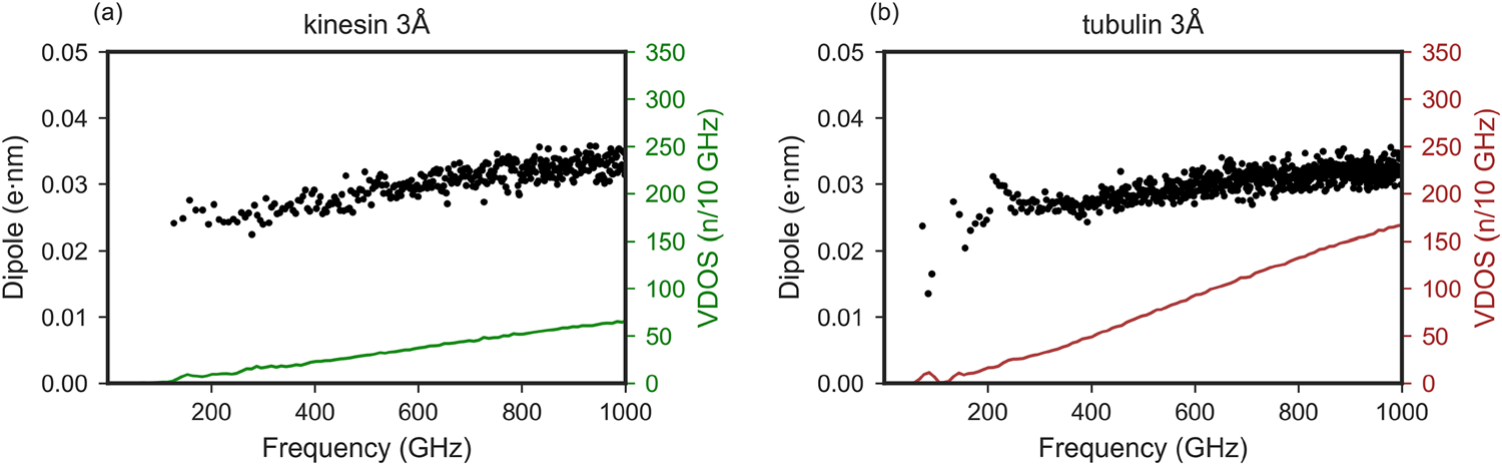
Comparison of variation of dipole of kinesin 3 Å and tubulin 3 Å systems, showing $\rho$ over frequency for first one thousand modes. Each dot in the figure represents the variation of dipole moment and frequency of a particular mode (averaged over 92 calculations). The green (**a**) and brown (**b**) curves indicate the number of modes per frequency bin for kinesin and tubulin system (bin size = 10 GHz).

**Fig. 7:**
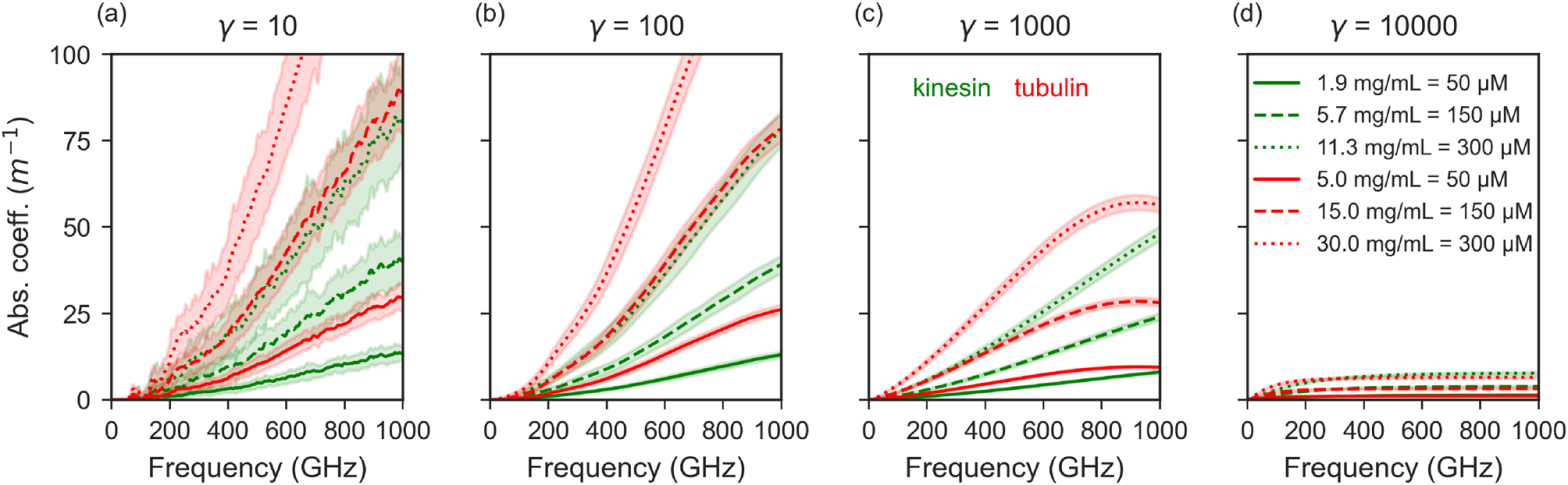
Absorption spectra of kinesin 3 Å and tubulin 3 Å systems: effect of concentration and damping. Absorption spectra corresponding to three concentrations 50 $\mu$M (kinesin 1.9 mg/mL, tubulin 5.0 mg/mL), 150 $\mu$M (kinesin 5.7 mg/mL, tubulin 15.0 mg/mL) and 300 $\mu$M (kinesin 11.3 mg/mL, tubulin 30.0 mg/mL) are shown as different line-styles. Kinesin and tubulin spectra are shown as green and red lines respectively. $\gamma$ is in GHz.

In our earlier study involving NMA of tubulin heterodimer, we had calculated the dielectric properties and absorption spectra in arbitrary units^54^. We had observed a strong bending/rotational motion of monomers with respect to each other for a few low-frequency vibrational modes. In the current study, we used the NMA-obtained eigenmodes of tubulin and calculated the variation of dipole moment and absorption spectra using the same method as mentioned above for the kinesin system to obtain results in absolute units. The tubulin 3 Å system showed much stronger absorption than the kinesin 3 Å system in the sub-THz region analyzed in this study (Fig. 7) for the damping coefficients $\gamma$ of 10 to 1000. At low damping, both systems exhibit distinct frequency-dependent absorption features that get weaker with higher damping. Comparison of the variation of the dipole of the first thousand eigenmodes with respect to frequency for the kinesin and tubulin systems shows that the values of $\rho$ are very similar for both systems (Fig. 6). The difference comes from the distribution of these modes over the frequency range. The first one thousand modes of tubulin span 50–1200 GHz, whereas the same number of modes for kinesin span 100–1800 GHz. This higher vibrational density of modes (VDOS) for tubulin results in a higher absorption by tubulin observed in Fig. 7. The higher VDOS in tubulin arises from the fact that the tubulin system contains more atoms – the VDOS of low-frequency modes of proteins scales as^57,58^ $g(\omega ) \propto N_f \,\omega$ with $N_f$ being degrees of freedom (that is $N_f=3N-6$, where N is the number of atoms) in the system (note that references^57,58^ used normalized-per-degree-of freedom VDOS $g_n(\omega _j) = \frac{n_j}{N_f \Delta \omega }$).

### Effect of protein concentration on absorption spectra

At low damping (Fig. 7a), both proteins exhibit broad and irregular absorption features, reflecting underdamped dynamics with pronounced resonance effects and sensitivity to local fluctuations. As $\gamma$ increases (100 - 1000 GHz), the spectra become smoother, with the absorption increasing more gradually with frequency. The relative magnitude of absorption also decreases, indicating reduced resonance sharpness and stronger dissipative effects. For a very strong damping ($\gamma$ = 10,000), the absorption spectra reach a nearly flat saturation profile at high frequencies and a broad absorption shoulder at low frequencies, characteristic of overdamped motion. The concentration dependence of proteins in aqueous solution has been experimentally investigated in multiple terahertz and sub-terahertz studies^59–64^. However, the specific contribution of protein vibrational modes to the overall absorption has rarely been analyzed, since the water solvent overwhelmingly dominates the absorption response, hence modified spectroscopic approaches have been used. In standard sub-THz-THz absorption spectroscopy, the bulk water, in particular its dipolar rotational degree of freedom, is the main absorber in the sub-THz range^44^. The water molecules in the first hydration shell of a protein are, however, strongly rotationally constrained and thus effectively “lost” from the sub-THz absorption^65^. By quantifying this reduction, one can estimate the number of water molecules within the protein’s hydration shell, an approach leveraged by several studies^65–67^. Once this effect is corrected for, it becomes possible to extract the absorption contribution arising from the vibrational modes of the proteins themselves^44,65^. This contribution clearly increases with protein concentration, as expected from the higher number of oscillators per unit volume. Nevertheless, it remains unclear whether this vibrational signature originates solely from collective internal vibrations of the protein atoms or also includes the coupled motion of the tightly bound hydration shell. In contrast to experimental works and earlier modelling works, our approach enables us to disentangle the respective contributions of the protein and its hydration envelope to the absorption due to vibrational degrees of freedom (see Fig. 4). In our calculations, the absorption scales nearly linearly with protein concentration, consistent with the proportional increase in the number of oscillators within the system (Fig. 7).

## Discussion of limitations and future work

Here we synthesized the main limitations and outline how results are expected to evolve in future work:

### Susceptibility and absorption in the context of water background

Figures 3, 4, 6, and 7 were obtained based on normal mode analysis. Consequently, these results include only the vibrational degrees of freedom and exclude any water background associated with other molecular motions (mainly rotational). To make a direct quantitative comparison with bulk or dry spectroscopy, one must either (1) apply an effective medium model that combines the protein and water permittivities, or (2) remove the non-vibrational contributions from experimental spectra before comparing them, as was attempted in^44,65^. For either approach, solution–phase predictions of $\alpha _{\textrm{mix}}(\nu )$ can be obtained using Maxwell–Garnett or Bruggeman mixing formulas with the measured $\varepsilon _{\textrm{water}}(\nu )$ and the same explicit protein number density $N_V$. A key difficulty is that a purely effective medium treatment is insufficient, since a portion of the surrounding water becomes dynamically restricted in the hydration shell and no longer contributes to the sub-THz response. This hydration-sequestration effect was estimated in Supplementary Information S1: a 3 Å hydration shell around kinesin contains $n_b = 1124$ water molecules per protein, leading to a removed-water fraction $f_{\textrm{rm}} \approx 0.0203\,c_{\textrm{mM}}$ (or $f_{\textrm{rm}}(\%) \approx 2.03\,c_{\textrm{mM}}$). For the experimental concentrations $c_{\textrm{mM}} = \{0.0516,\,0.155,\,0.307,\,0.517\}$ (corresponding to 1.9–19 mg $\hbox {mL}^{-1}$), the resulting fractions are $f_{\textrm{rm}} = \{0.105,\,0.315,\,0.623,\,1.050\}\%$. Using a dilute linear-mixing approximation with the complex permittivity of water at 100 and 200 GHz^68,69^ (consistent with model and experimental intercomparisons^70,71^), the bound-water effect reduces $\chi '$, $\chi ''$, and $\alpha$ approximately in proportion to $f_{\textrm{rm}}$. At the highest concentration (0.517 mM), we estimate upper-bound decreases of $\Delta \chi '(100 \mathrm {\,GHz})\!\approx \!-0.083$, $\Delta \chi ''(100 \mathrm {\,GHz})\!\approx \!-0.146$, and $\Delta \alpha _{\mathrm {\,GHz}}\!\approx \!-8.6\times 10^{1}\,\mathrm {m^{-1}}$, with a similar scaling at 200 GHz. Since hydration water retains a reduced and spectrally shifted response^65^, these values represent conservative upper limits. Finally, geometric overlap of 3 Å hydration shells occurs only near $c\!\sim \!0.3-0.5~\mathrm {g\,mL^{-1}}$, well above our range, so inter-shell effects can be neglected (see SI, Sec. S1). This back-of-the-envelope estimate illustrates a practical way to link modelling results with experimental data. 

### Electronic polarization

Our mode dipole variations ${}^{m}\!\rho _l$ are evaluated using fixed partial charges and therefore capture the nuclear (ionic) polarization associated with normal-mode displacements, but they do not include explicit induced dipoles (electron-cloud deformation). On the time scale of nuclear motion, electronic polarization responds essentially instantaneously and is commonly represented by a high-frequency permittivity $\varepsilon _\infty =\varepsilon _{\textrm{el}}=n^2\approx 2$ for organic media^72^ and proteins^73^. Including such a baseline would shift $\varepsilon _r'(\nu )$ by an approximately frequency-independent constant in the sub-THz band, while the frequency-dependent loss $\varepsilon _r''(\nu )$ remains governed by the vibrational modes. In the weak-loss limit ($\varepsilon _r''\ll \varepsilon _r'$), Eq. (6) implies $\alpha (\nu )\propto \varepsilon _r''(\nu )/\sqrt{\varepsilon _r'(\nu )}$, so adding $\varepsilon _\infty$ would mainly rescale $\alpha$ by a factor $\sim \!\left[ \varepsilon _r'/( \varepsilon _r'+\varepsilon _\infty )\right] ^{1/2}$, without altering the relative hydration-induced blue-shifts or the protein–water cancellation trends emphasized here. A more complete quantification of induced polarization effects could be obtained in future work by repeating the analysis with a polarizable force field (e.g., classical Drude or induced-dipole models)^74^.

### Harmonic modes, hydration, and damping

Our spectra were built from harmonic normal modes of energy-minimized structures with either no or 3 Å hydration shell. This isolated the bound-water regime suggested by our MD persistence analysis (Fig. 2) and kept the computation tractable, but it omitted anharmonicity, energy dissipation, as well as long-range hydration that can shift or damp sub-THz modes^19,59,60,75–77^. To reduce sensitivity to conformational heterogeneity, we employed an *ensemble* of NMA calculations over MD-sampled snapshots—akin to normal-mode ensemble analysis (NMEA)^19^, which provided a better representation by a thorough sampling of contributions from the protein’s conformational phase-space. If only a single energy-minimized crystal structure is used for NMA^15,35^, the generality of the spectral findings would be uncertain. Furthermore, to assess sensitivity to dissipation, we also swept a single phenomenological damping rate across four decades ($\gamma =10\ldots 10^{4}$ GHz); encouragingly, the qualitative hydration trends are robust to this choice of $\gamma$. We emphasize that using a uniform $\gamma$ does not imply identical linewidths or quality factors across all modes in real experimental protein system; different modes are likely governed by distinct dissipative mechanisms. In our current work, $\gamma$ serves here as an *effective* homogeneous broadening parameter to probe the robustness of hydration-induced trends across underdamped-to-overdamped regimes. In addition, by averaging NMA over 92 MD-sampled conformations, we already incorporate a degree of *inhomogeneous* broadening via the dispersion of $\nu _l$ and $\rho _l$ across snapshots, conceptually similar to ensemble ENM/NMA approaches^19,78^ that broaden mode frequencies by analyzing multiple MD snapshots. As future work, we plan to (i) complement the present NMA framework with explicit-solvent, periodic MD combined with Green–Kubo (linear-response, fluctuation–dissipation) calculations of dielectric spectra^79–81^, and (ii) *map normal-mode coordinates onto MD trajectories* to extract realistic frequency distributions and effective mode-specific damping, either by projecting NMA modes onto explicit-solvent MD trajectories or by identifying frequency-selective modes directly from MD velocities, as in the FRESEAN approach^75^. The explicit-solvent calculations and MD-mapped mode analyses are substantial and beyond the scope of the present manuscript.

### Protein system selection implications

In this work, we analyzed only the catalytic motor domain of kinesin-1 (KIF5B) to isolate its intrinsic sub-THz vibrational dielectric response from the effects of distant domains and neighboring proteins. Kinesin attains full activity on microtubules (MTs), which regulate nucleotide exchange and stepping kinetics^41^. From a spectroscopic and physical standpoint, omitting the covalently linked neck linker, coiled-coil, and tail chiefly alters long-range elastic couplings that can shift or split the softest modes; these distal elements make a minor contribution to dipole-active modes of the motor domain, so their exclusion retains the dominant signatures of the motor core, we believe. Skipping the non-covalent neighbor (tubulin) removes interfacial mechanical constraints that would create inter-protein collective modes. The present spectra should therefore be read as a reference for the hydrated, isolated motor domain. Larger assemblies (dimeric kinesin engaged with the MT lattice) may be approached with coarse-grained strategies that recover global modes at tractable cost, such as elastic network model (ENM)-based and related toolchains^7,82,83^ or finite-element coarse-grained frameworks^46^. However, these coarse-grained models omit explicit solvent; consequently, they cannot capture the hydration-shell network that shapes susceptibility and absorption (including the non-additivity and blue-shifts we quantify). For this reason, while coarse-grained ENM-based models are promising for large system size, they cannot answer the spectroscopic questions posed in this paper.

Although the primary focus of this study is on the influence of hydration on vibrational modes and their electromagnetic properties, we have performed an additional residue-level displacement analysis for selected normal mode eigenvectors of the kinesin motor domain to clarify the structural contributions to low-frequency vibrations. This supplementary analysis provides structural context for the collective motions captured by the low-frequency modes. Specifically, per-residue RMS displacement profiles were computed for representative low-, intermediate-, and high-frequency eigenvectors, highlighting the relative involvement of distinct secondary-structure elements, including regions implicated in the kinesin–tubulin interface. The methodology and detailed results of this analysis are presented in Supplementary Information Sec. S1–2 and Fig. S1–2.

## Conclusion

This study provides a quantitative molecular-level picture of how hydration modulates the sub-THz vibrational component of the dielectric response of the kinesin motor domain. Using molecular dynamics simulations combined with normal mode analysis, we computed low-frequency vibrational modes, their frequencies, and dipole moment variations, and predicted absorption spectra in absolute units. Based upon dynamics of water molecules in different water shells, water molecules within 3 Å around the protein were considered bound-water and incorporated in NMA calculations. Including the 3 Å water layer in the system leads to a blue-shift and reduced amplitude in the absorption spectra. The decomposition of protein and water contributions demonstrated non-additive absorption arising from partially antiparallel dipole variations, highlighting the importance of protein–water dynamical coupling in shaping sub-THz response. We also compared kinesin and tubulin systems and showed the effects of concentration and damping on absorption spectra. The comparison of kinesin and tubulin systems also showed that differences in mode density strongly influence sub-THz absorption strength. Together, these results provide insight into how hydration and vibrational mode properties modulate electrodynamic properties of proteins.

## Methods

### Structure preparation and normal mode analysis (NMA)

The initial structures of kinesin used here were taken from our previous study^48^ in which three independent molecular dynamics simulations of the kinesin–tubulin complex were performed, each with a 30 ns long production run. The stability of kinesin conformations were confirmed using root mean square deviation (RMSD) analysis, see Fig. S1-1 and text in Supplementary Information S1, Sec. S1-1). The structures of kinesin (Fig. 1) bound with ADP and Mg molecules were extracted from these trajectories, each separated by 1 ns time interval. Two types of systems were prepared: protein-ADP-Mg complex, Protein-ADP-Mg with 3 Å water layer. Each type of system here had 92 structures. CHARMM36 all-atom force field^84^ was used to define the charges and parameters for protein and ligand. For normal mode analysis (NMA), it is necessary to use fully energy-minimized structures of protein molecules. In order to achieve this, the systems were energy minimized in two steps: first using steepest descent and then l-bfgs methods in Gromacs^85,86^. Here the mdrun program was used in double precision. For the calculation of long-range interactions reaction-field method was used with a cut-off value of 1.2 nm and rcoulomb-switch at 1 nm. vdW interactions were computed using Potential-switch and rvdw-switch of 1 nm and rvdw cut-off 1.2 nm. After the systems were fully energy minimized, the final conformations were used to generate hessian matrices using nm integrator in Gromacs. The hessian matrices were diagonalized using nmeig tool in gromacs in order to obtain eigenmodes and their corresponding frequencies. Ninety two such calculations were done for both types of system (with and without water), and the first one thousand eigenmodes along with their frequencies were extracted.

### Dielectric susceptibility and absorption due to protein normal vibrational modes

We model a vibrational dielectric response of a protein as a sum of independent, damped harmonic modes that carry a time-varying dipole moment and couple linearly to an external electric field. See details of assumptions and all derivations in SI 2. For a mode $l$ with center frequency $\nu _l$ (Hz) and damping rate $\gamma _l$ (s$^{-1}$), the driven steady-state dipole response yields a Lorentz-form contribution to the complex susceptibility. For a dilute, isotropic ensemble probed by a linearly polarized field, orientational averaging contributes the standard factor $1/3$. The complex susceptibility (sum over $M$ modes) is

1 $$\begin{aligned} \chi (\nu ) \;=\; \frac{1}{3}\,\frac{N_V}{\varepsilon _0\,4\pi ^2}\, \sum _{l=1}^{M}\frac{\bigl ({}^{m}\!\rho _l\bigr )^2}{\nu _l^{2}-\nu ^{2}-j\,\nu \,\gamma _l}, \end{aligned}$$

where $N_V$ is the molecular number density (m$^{-3}$) and $\varepsilon _0$ is the vacuum permittivity. The mass-weighted effective charge (dipole derivative) of mode $l$ is

2 $$\begin{aligned} {}^{m}\!\rho _l \;=\; \left\| \sum _{i} q_i\,\textbf{e}_{l,i} \right\| , \end{aligned}$$

computed from per-atom partial charges $q_i$ and GROMACS normal-mode eigenvectors $\textbf{e}_{l,i}$ (Cartesian output with units kg$^{-1/2}$); $\Vert \cdot \Vert$ denotes the Euclidean norm. Mode wavenumbers $\tilde{\nu }$ (cm$^{-1}$) are converted to Hz via $\nu = (2.997\,924\,58\times 10^{10})\,\tilde{\nu }$.

Separating real and imaginary parts with $A_l(\nu )=\nu _l^2-\nu ^2$ and $B_l(\nu )=\nu \,\gamma _l$,

3 $$\begin{aligned} \chi '(\nu ) \;=\; \frac{N_V}{\varepsilon _0\,12\pi ^2}\, \sum _{l=1}^{M} \frac{\bigl ({}^{m}\!\rho _l\bigr )^2\,A_l}{A_l^2+B_l^2}, \end{aligned}$$

4 $$\begin{aligned} \chi ''(\nu ) \;=\; \frac{N_V}{\varepsilon _0\,12\pi ^2}\, \sum _{l=1}^{M} \frac{\bigl ({}^{m}\!\rho _l\bigr )^2\,B_l}{A_l^2+B_l^2}. \end{aligned}$$

The complex relative permittivity is $\varepsilon _r(\nu )=1+\chi (\nu )=\varepsilon _r'(\nu )+j\,\varepsilon _r''(\nu )$ with $\varepsilon _r' = 1+\chi '$ and $\varepsilon _r''=\chi ''$. For a non-magnetic medium, writing the complex refractive index as $\tilde{n}=n+jk$ and using $\tilde{n}^2=\varepsilon _r$, the extinction coefficient is

5 $$\begin{aligned} k(\nu )=\sqrt{\frac{1}{2}\!\left( -\varepsilon _r'(\nu )+\sqrt{\varepsilon _r'^2(\nu )+\varepsilon _r''^2(\nu )}\right) }. \end{aligned}$$

The absorption coefficient follows as

6 $$\begin{aligned} \alpha (\nu )=\frac{4\pi \nu }{c}\,k(\nu ), \end{aligned}$$

where $c$ is the speed of light.

The practical pipeline for the calculation was as follows: (i) Perform GROMACS normal-mode analysis to obtain $\nu _l$ and eigenvectors $\textbf{e}_{l,i}$ (choose $\gamma _l$ phenomenologically as a parameter). (ii) Compute ${}^{m}\!\rho _l$ from $q_i$ and $\textbf{e}_{l,i}$. (iii) Set $N_V$ from concentration via Avogadro’s number. (iv) Evaluate $\chi '(\nu )$ and $\chi ''(\nu )$, then $\varepsilon _r'(\nu )$, $\varepsilon _r''(\nu )$, and $\alpha (\nu )$. (v) Calculate the average of the spectra across the ensemble of all 92 replicas of molecular systems. Our assumptions and scope include: linear response; independent modes (no inter-mode coupling); phenomenological damping; isotropic orientation average; dilute, non-interacting molecules.

## Supplementary Information

Supplementary Information 1.

Supplementary Information 2.

## Data Availability

Kinesin NMA data: https://hdl.handle.net/11104/0371525281. Tubulin NMA data: https://doi.org/10.57680/asep.0584789282. Kinesin MD simulations trajectories: https://doi.org/10.5281/zenodo.2644158283.
