# Supplementary material for: Vibrational contribution to the sub-terahertz dielectric response of kinesin and its hydration shell
Source: Sci Rep. 2026 Mar 1;16:11508. doi: 10.1038/s41598-026-40625-0 (PMC13056937; doi:10.1038/s41598-026-40625-0)
Supplement: Supplementary file 1 — Supplementary Information 1. [file 41598_2026_40625_MOESM1_ESM.pdf]

Supplementary information 1 for ”Vibrational Contribution to the Sub-Terahertz Dielectric Response of Kinesin and Its Hydration Shell”

Saurabh K. Pandey[1] and Michal Cifra[1,*]

[1]Institute of Photonics and Electronics of the Czech Academy of Sciences, Prague, Czechia
[*]cifra@ufe.cz

ABSTRACT

-

S1-1 Molecular dynamics simulation trajectories stability analysis

The data used in the present work are derived from a previously published study[1] in which three independent molecular dynamics simulations of the kinesin–tubulin complex were performed, each with a 30 ns long production run. The kinesin structures together with their hydration shells were extracted from these trajectories and used for the analyses presented here. RMSD was calculated for the Cα atoms using the energy-minimized structure as a reference. Figure S1-1 shows root mean square deviation (RMSD) of three trajectories, each 30 ns long. The trajectories show a flat profile with a mean value of approximately 1.5 $\AA$. The fluctuations in the RMSD values are within 1 $\AA$ which shows that systems are equilibrated and stable. The comparable RMSD profiles across the three simulations indicate consistent structural stability and reproducibility of the kinesin dynamics under the simulated conditions.

S1-2 Eigenvectors displacement analysis

To quantify residue-level flexibility of the kinesin motor domain and to identify which secondary structure elements are involved in particular normal modes, a few eigenvectors obtained from NMA of a representative kinesin-3$\AA$ system were analyzed. For each eigenvector, atom-wise displacement magnitudes were calculated as

$$|\Delta \mathbf{r}_i| = \sqrt{(\Delta x_i)^2 + (\Delta y_i)^2 + (\Delta z_i)^2} \tag{S1-1}$$

where Δx_i, Δy_i and Δz_i are the cartesian displacement components of atom i of an eigenvector. Only protein atoms belonging to residues 1–325 of the kinesin motor domain were included in the analysis. The first six modes, corresponding to overall translational and rotational motions, were excluded. To obtain displacement value per residue, atom-wise eigenvector components were aggregated using a root-mean-square (RMS) measure as

$$\mathrm{RMS}_r = \sqrt{\frac{1}{n_r}\sum_{i\in r} |\Delta \mathbf{r}_i|^2} \tag{S1-2}$$

where n_r is number of atoms in a residue.

Figure S1-2 shows the analysis of the displacement profiles per-residue for selected eigenvectors, revealing distinct patterns of mobility associated with specific secondary structure elements. We included displacement profiles for representative low- (panel a-b), intermediate- (panel c-d), and high-frequency eigenvectors (panel e), grouped into five panels for clarity. In the low-frequency eigenvectors (eigenvectors 1–12; Figure S1-2a-b), the largest displacement amplitudes are concentrated in the loops (L6, L10), nucleotide-sensitive Switch I (loop L9) and Switch II (loop L11) regions, as well as in surface-exposed microtubule-interacting loops such as L8 and L12. In contrast, the central β-sheet and core α-helices exhibit comparatively reduced mobility, consistent with their role as a rigid mechanical scaffold. Intermediate-frequency eigenvectors (eigenvectors 13–24) display progressively more localized motions of smaller amplitude (Figure S1-2c–d). In the highest-frequency eigenvectors examined (eigenvectors 989–994; Figure S1-2e), displacement amplitudes are uniformly small and spatially dispersed across the structure, indicating localized, high-energy fluctuations that are unlikely to contribute directly to large-scale functional transitions.

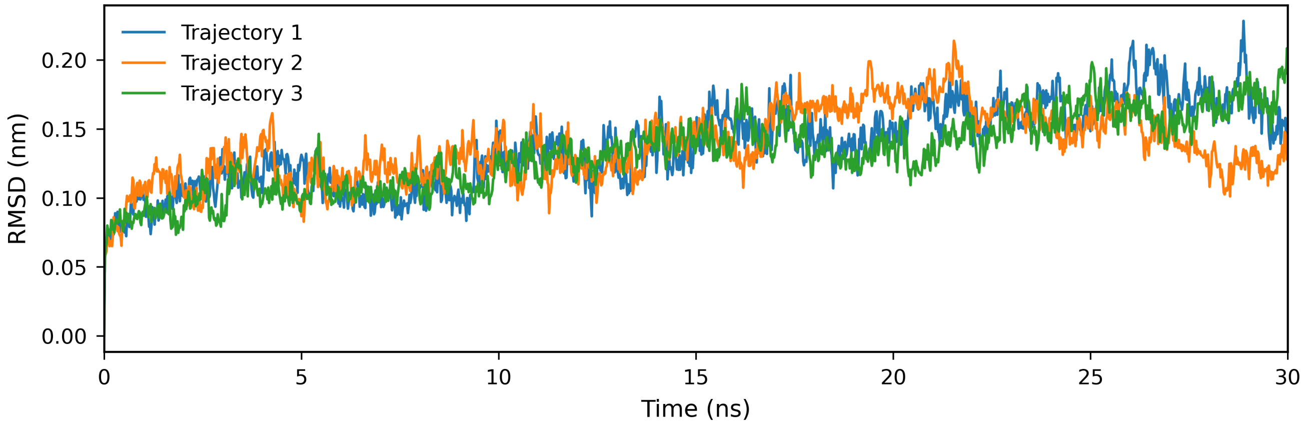

Figure S1-1. Root mean square deviation (RMSD) of the kinesin for three independent molecular dynamics simulations, each of 30 ns duration, shown as blue, orange , and green lines. RMSDs were calculated for the Cα atoms of kinesin residues with energy-minimized structure used as a reference.

S1-3 Dipole Variation and Absorption Behavior of Kinesin and Tubulin under Hydration and Damping Effects

Figures S1-3–S1-5 summarize the variation of mode-resolved dipole moments and the corresponding absorption characteristics for kinesin and tubulin systems under different hydration and damping conditions. Figure S1-3 compares the first ten vibrational modes of kinesin in the dry (0 Å) and hydrated (3 Å) states, showing that hydration slightly reduces the average dipole strength while preserving the overall frequency distribution. Each colored dot represents a single mode from one of the 92 molecular models, whereas the larger outlined circles denote the mean dipole values across all models for a given mode. Extending the analysis to the first thousand modes, Figure S1-4 displays the dipole variation for kinesin and tubulin systems with a 3 Å hydration shell. The scatter plots reveal a broad spread of dipole magnitudes with frequency, while the overlaid mode-count curves indicate that tubulin exhibits a higher density of modes in the sub-terahertz region compared with kinesin. This suggests that tubulin possesses a richer distribution of low-frequency collective motions contributing to its dielectric response. Finally, Figure S1-5 presents the computed absorption spectra of hydrated kinesin and tubulin for different damping factors ($\gamma = 10$–10,000 GHz). The spectra exhibit a monotonic increase in absorption with both frequency and damping. Tubulin consistently shows higher absorption coefficients than kinesin, reflecting its larger dipole variability and greater mode density. Together, these results demonstrate how hydration and molecular damping jointly modulate the protein-level dielectric and absorptive behavior across the gigahertz-to-terahertz frequency range.

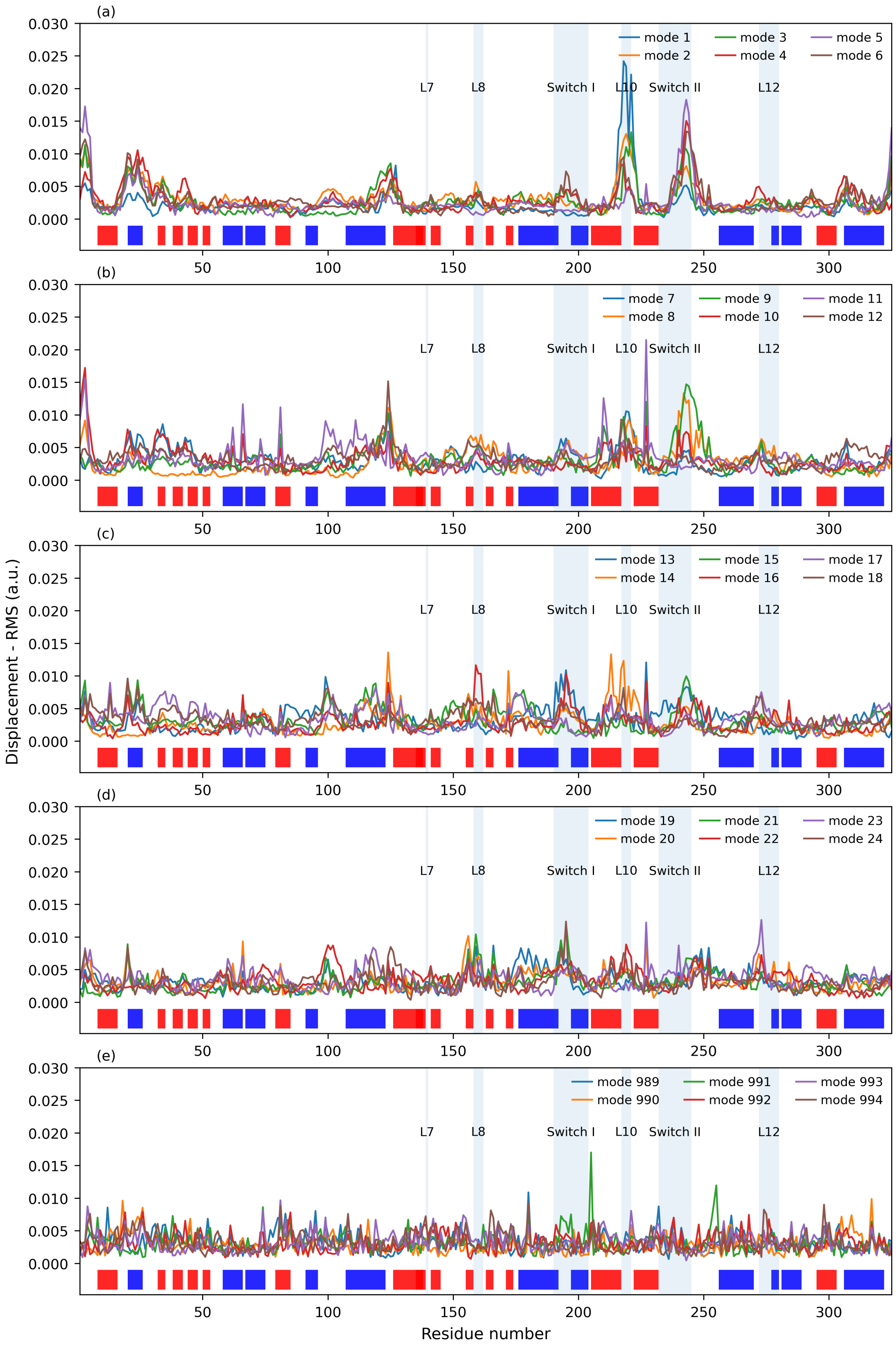

Figure S1-2. Per-residue displacement profiles of kinesin motor domain from normal mode analysis: Per-residue root mean square (RMS) displacement profiles are shown for selected eigenvectors obtained from NMA of a representative snapshot for the kinesin motor domain. The first six modes corresponding to global translational and rotational motions were excluded. Panels (a–e) show groups of eigenvectors: (a) eigenmodes 1–6 (b) eigenmodes 7–12 (c) eigenmodes 13–18 (d) eigenmodes 19–24 , and (e) eigenmodes 989–994. The x-axis represents residue number, and the y-axis represents RMS displacement (arbitrary units). Secondary structure elements are indicated below each panel, with α-helices shown in blue and β-strands shown in red. Peaks in the displacement profiles highlight regions undergoing larger collective motions, while low-amplitude regions indicate structurally rigid segments.

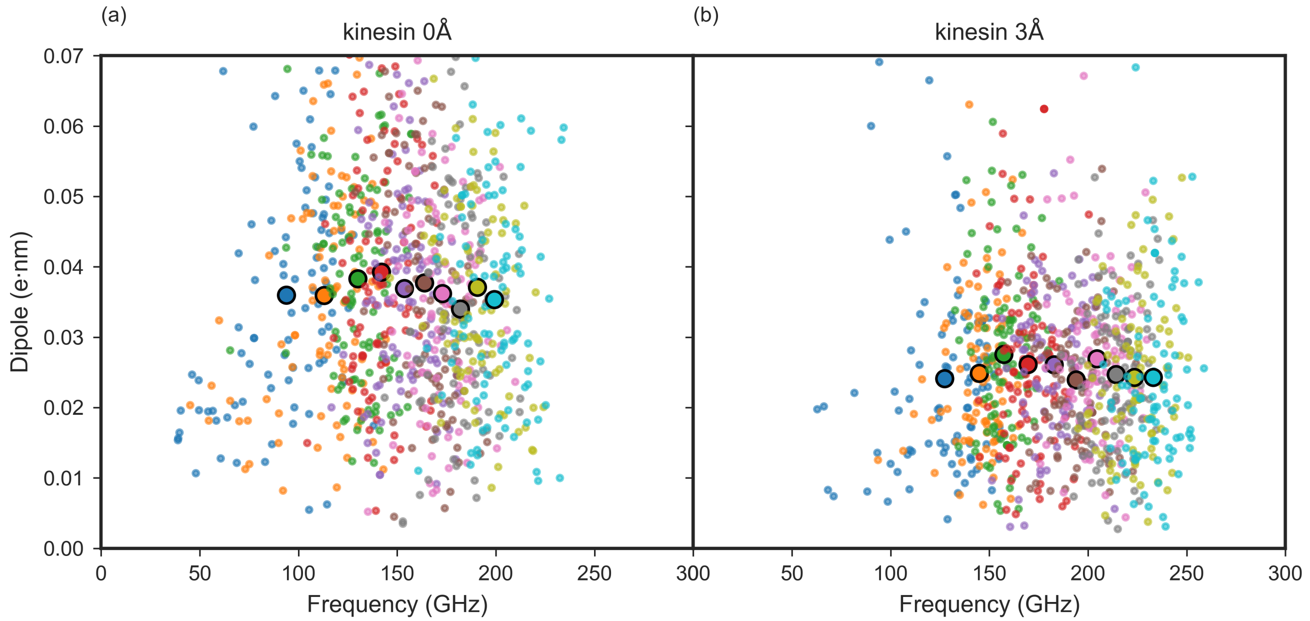

Figure S1-3. Comparison of variation of dipole of kinesin 0 Å and kinesin 3 Å system, showing ρ over frequency for first ten modes. Each small dot represents the dipole moment and frequency of a given mode from one of the 92 models, while the larger outlined circles indicate the average values over all models for each mode.

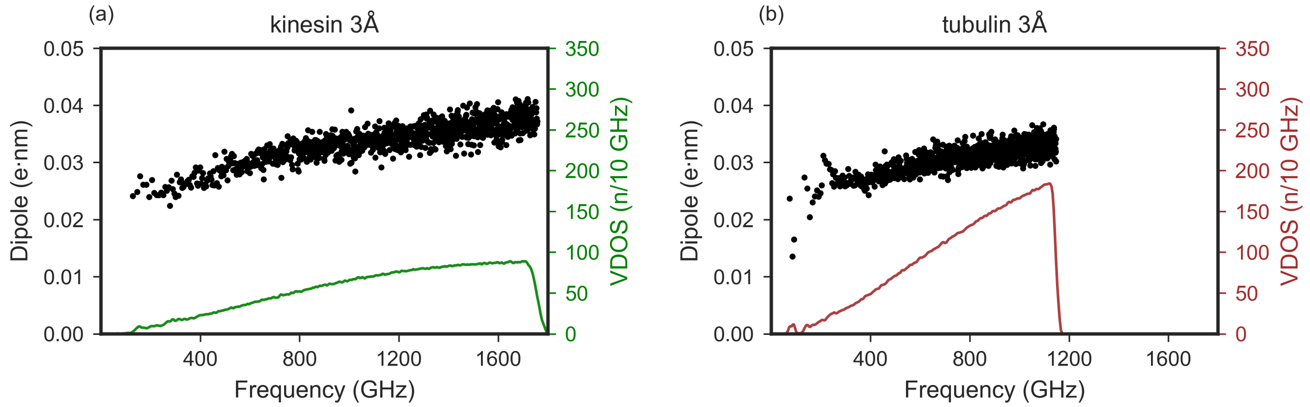

Figure S1-4. Comparison of variation of dipole of kinesin and tubulin systems (with 3 Å water), showing ρ over frequency for first one thousand modes. Each dot in the figure represent variation of dipole moment and frequency of a particular mode (averaged over 92 calculations). The green and brown curves indicate the number of modes per frequency bin for kinesin and tubulin system(bin size = 10 GHz).

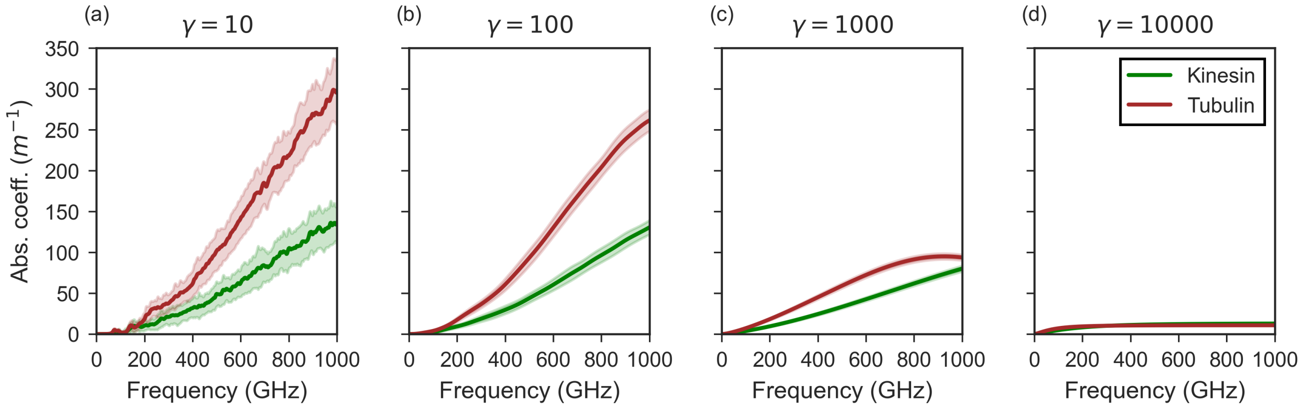

Figure S1-5. Comparison of absorption spectra of kinesin 3 Å and tubulin 3 Å, effect of damping. The concentrations of kinesin and tubulin are 1.9 mg/mL and 5.0 mg/mL, having matching molar concentration of 50 μM

S1-4 Hydration–sequestered water and its impact on χ and α at 100/200 GHz

To estimate how much water is rendered "bound" (and thus effectively removed from bulk THz absorption) by kinesin at the concentrations used, we use the explicit count from our model: the 3 Å hydration shell around kinesin contains $n_b = 1124$ water molecules per protein. For comparison, a solvent–accessible surface area (SASA) estimate using $A_{\text{kinesin,SASA}} = 173\ \text{nm}^2$ would yield $\sim 1.73 \times 10^3$ waters.

We work in millimolar (mM) units for clarity. Since 1 mM = 1 mol m^{-3}, the protein number density is simply

$$N_V = c_{\text{mM}} N_A. \tag{S1-3}$$

With bulk-water number density $N_{\text{w}} \approx 55.5 \times 10^3 N_A \simeq 3.34 \times 10^{28}\ \text{m}^{-3}$, the "removed–water" fraction becomes

$$f_{\text{rm}} \approx \frac{n_b N_V}{N_{\text{w}}} = \left(\frac{n_b N_A}{N_{\text{w}}}\right) c_{\text{mM}} \simeq 0.0203\, c_{\text{mM}}, \tag{S1-4}$$

i.e. $f_{\text{rm}}(\%) \simeq 2.03\, c_{\text{mM}}$.

For the four working concentrations

$$c_{\text{mM}} = \{0.0516,\ 0.155,\ 0.307,\ 0.517\}\ \text{mM} \quad \big(\text{which correspond to } \{1.9,\ 5.7,\ 11.3,\ 19\}\ \text{mg mL}^{-1}\big),$$

Eq. (S1-4) gives

$$f_{\text{rm}} \approx \begin{cases} 0.105\% & c_{\text{mM}} = 0.0516, \\ 0.315\% & c_{\text{mM}} = 0.155, \\ 0.623\% & c_{\text{mM}} = 0.307, \\ 1.050\% & c_{\text{mM}} = 0.517. \end{cases}$$

Relation between susceptibility, refractive index, and absorption

Adopting the $e^{-j\omega t}$ convention, we write

$$\chi(\nu) = \chi'(\nu) + j\chi''(\nu), \qquad m = n + j\kappa = \sqrt{\varepsilon_r}, \qquad \alpha = \frac{4\pi f \kappa}{c}. \tag{S1-5}$$

For water near room temperature we use

$$\varepsilon_r(100\ \text{GHz}) \approx 8.9 + j14.0, \qquad \varepsilon_r(200\ \text{GHz}) \approx 5.6 + j7.0, \tag{S1-6}$$

from a double–Debye description of liquid water[2,3]. Hence,

$$\chi_{\text{w}}(100) \approx 7.9 + j14.0, \quad \chi_{\text{w}}(200) \approx 4.6 + j7.0,$$

and

$$\alpha_{\text{water}}(100\ \text{GHz}) \approx 8.22 \times 10^3\ \text{m}^{-1}, \qquad \alpha_{\text{water}}(200\ \text{GHz}) \approx 1.09 \times 10^4\ \text{m}^{-1}.$$

These values are consistent with broadband model–measurement intercomparisons in the 31–225 GHz range[4,5].

Bound–water effect as linear mixing

Approximating the "missing–water" effect by dilute linear mixing gives

$$\Delta\chi' \approx -f_{\rm rm}\,\chi'_{\rm w}, \qquad \Delta\chi'' \approx -f_{\rm rm}\,\chi''_{\rm w}, \qquad \Delta\alpha \approx -f_{\rm rm}\,\alpha_{\rm water}. \tag{S1-7}$$

The computed reductions (negative values) are summarized in Table S1-1.

Table S1-1. Estimated reductions in χ' and χ'' and absorption coefficient α due to bound water for kinesin concentrations in mM (corresponding mg/mL in parentheses).

	$f_{\rm rm}$ (%)	$\Delta\chi'(100)$	$\Delta\chi''(100)$	$\Delta\alpha_{100}$ [m^{-1}]	$\Delta\chi'(200)$	$\Delta\chi''(200)$	$\Delta\alpha_{200}$ [m^{-1}]
$c = 0.0516$ mM (1.9 mg mL^{-1})	0.105	-0.0083	-0.0147	-8.63×10^{0}	-0.0048	-0.0073	-1.14×10^{1}
$c = 0.155$ mM (5.7 mg mL^{-1})	0.315	-0.0249	-0.0441	-2.59×10^{1}	-0.0145	-0.0221	-3.43×10^{1}
$c = 0.307$ mM (11.3 mg mL^{-1})	0.623	-0.0491	-0.0870	-5.11×10^{1}	-0.0286	-0.0435	-6.73×10^{1}
$c = 0.517$ mM (19 mg mL^{-1})	1.050	-0.0830	-0.146	-8.59×10^{1}	-0.048	-0.073	-1.14×10^{2}

These values scale linearly with the assumed shell thickness and n_b. Because bound water retains a diminished and shifted dielectric response[6], the numbers above represent upper bounds on the reduction to the bulk–water contribution in χ and α at 100–200 GHz. Permittivity and absorption values are taken from double–Debye fits to liquid water[2,3].

When do 3 Å hydration shells begin to overlap?

Using again $n_b = 1124$ waters per protein and the number density of bulk water $N_{\rm w}^{\rm (num)} \approx 33.4$ nm^{-3}, the shell volume is

$$V_{\rm shell} \approx \frac{1124}{33.4} \simeq 33.6\ {\rm nm}^3. \tag{S1-8}$$

With specific volume $\bar{v} \approx 0.73$ mL g^{-1} and $M = 36.8$ kDa, the protein volume is

$$V_{\rm prot} \approx 44.6\ {\rm nm}^3. \tag{S1-9}$$

The combined 3 Å-expanded (core + shell) "exclusion" volume is

$$V_{3\text{Å}} \approx 78.2\ {\rm nm}^3, \qquad r_{\rm eff} \approx 2.65\ {\rm nm}. \tag{S1-10}$$

A touching criterion $d \approx 2r_{\rm eff}$ implies

$$N_V^{\rm (touch)} \approx \frac{1}{(2r_{\rm eff})^3} \simeq 6.7\times10^{24}\ {\rm m}^{-3}, \qquad c_{\rm overlap}^{\rm (touch)} \approx 0.41\ {\rm g\,mL}^{-1}. \tag{S1-11}$$

Bracketing by volume fraction $\phi = N_V V_{3\text{Å}}$, we obtain:

$$\text{Continuum percolation (overlapping spheres), } \phi_c \approx 0.289^{7}: \quad c \approx 0.29\,\frac{M/N_A}{V_{3\text{Å}}} \simeq 2.3\times10^2\ {\rm mg\,mL}^{-1}, \tag{S1-12}$$

$$\text{Random close packing (hard spheres), } \phi \approx 0.64^{8}: \quad c \approx 0.64\,\frac{M/N_A}{V_{3\text{Å}}} \simeq 5.0\times10^2\ {\rm mg\,mL}^{-1}, \tag{S1-13}$$

$$\text{Space filling, } \phi = 1: \quad c \approx \frac{M/N_A}{V_{3\text{Å}}} \simeq 7.8\times10^2\ {\rm mg\,mL}^{-1}. \tag{S1-14}$$

Using the explicit $n_b = 1124$ waters in the 3 Å shell, hydration shells begin to geometrically overlap at concentrations of order

$$c \sim 0.3\text{–}0.5\ {\rm g\,mL}^{-1}, \quad \text{central estimate: } c_{\rm overlap} \approx 0.41\ {\rm g\,mL}^{-1}.$$

Our working concentrations 0.051–0.517 mM (i.e. 1.9–19 mg mL^{-1}) are well below this range, so shell–shell overlap should be rare under those conditions.

References

1. Průša, J. & Cifra, M. Molecular dynamics simulation of the nanosecond pulsed electric field effect on kinesin nanomotor. *Scientific Reports* **9**, 19721 (2019).

2. Meissner, T. & Wentz, F. The complex dielectric constant of pure and sea water from microwave satellite observations. *IEEE Transactions on Geoscience and Remote Sensing* **42**, 1836–1849 (2004).

3. Ellison, W. J. Permittivity of Pure Water, at Standard Atmospheric Pressure, over the Frequency Range 0–25THz and the Temperature Range 0–100°C. *Journal of Physical and Chemical Reference Data* **36**, 1–18 (2007).

4. Kneifel, S. *et al.* Absorption Properties of Supercooled Liquid Water between 31 and 225 GHz: Evaluation of Absorption Models Using Ground-Based Observations. *Journal of Applied Meteorology and Climatology* **53**, 1028–1045 (2014).

5. Turner, D. D., Kneifel, S. & Cadeddu, M. P. An Improved Liquid Water Absorption Model at Microwave Frequencies for Supercooled Liquid Water Clouds. *Journal of Atmospheric and Oceanic Technology* **33**, 44 (2016).

6. Charkhesht, A., Regmi, C. K., Mitchell-Koch, K. R., Cheng, S. & Vinh, N. Q. High-Precision Megahertz-to-Terahertz Dielectric Spectroscopy of Protein Collective Motions and Hydration Dynamics. *The Journal of Physical Chemistry B* **122**, 6341–6350 (2018).

7. Torquato, S. & others. *Random heterogeneous materials: microstructure and macroscopic properties*, vol. 16 (Springer, 2002).

8. Bernal, J. D. The Bakerian lecture, 1962. The structure of liquids. *Proceedings of the Royal Society of London. Series A, Mathematical and Physical Sciences* **280**, 299–322 (1964). Publisher: JSTOR.
