# Supplementary material for: Vibrational contribution to the sub-terahertz dielectric response of kinesin and its hydration shell
Source: Sci Rep. 2026 Mar 1;16:11508. doi: 10.1038/s41598-026-40625-0 (PMC13056937; doi:10.1038/s41598-026-40625-0)
Supplement: Supplementary file 2 — Supplementary Information 2. [file 41598_2026_40625_MOESM2_ESM.pdf]

Supplementary information 2 for "Vibrational Contribution to the Sub-Terahertz Dielectric Response of Kinesin and Its Hydration Shell"

Saurabh K. Pandey, Michal Cifra

January 24, 2026

Contents

S2-1 Introduction . . . 1
S2-2 From Complex Permittivity to Absorption for Nonmagnetic Materials . . . 2
S2-3 From the Lorentz Oscillator to Permittivity and Absorption for Single Mode . . . 3
S2-4 Real and Imaginary Part of Permittivity and Absorption . . . 3
S2-5 Effective Charge (Dipole Variation) and How to Obtain it from Modal Eigenvectors and Partial Charges of Atoms . . . 4
S2-6 Generalization to Multiple Modes . . . 5
S2-7 Conversion of Chemical Units to Number Density . . . 5
S2-8 Limitations of the NMA Approach for Calculating Absorption . . . 6
S2-9 Appendix: Clarification on GROMACS NMA eigenvectors: notation, properties, and units . . . 6
S2-9.1 What GROMACS outputs (`gmx nmeig`). . . . 7
S2-9.2 Orthogonality and normalization. . . . 7
S2-9.3 Units / dimensions. . . . 7
S2-9.4 Practical note on GROMACS option. . . . 7
S2-10 Summary of Units and Dimensions . . . 7
S2-11 References . . . 9

S2-1 Introduction

Biological macromolecules such as proteins can be modeled as *multimodal mechanical oscillators* carrying a complex distribution of electric charge. Each normal vibrational mode corresponds to a collective motion of atoms and is approximated as a harmonic oscillator. Because proteins contain charged or polar groups, these vibrational motions are accompanied by a time-varying dipole moment, enabling coupling to external electromagnetic fields. This document develops the general equations for the frequency-dependent complex permittivity and absorption of such systems within the framework of classical linear response theory. It also clarifies the units of the various quantities used, and discusses the normalization of eigenvectors in normal mode analysis, particularly as obtained from Gromacs.

S2-2 From Complex Permittivity to Absorption for Nonmagnetic Materials

The complex relative permittivity is defined as:

$$\epsilon_r(\nu) = \epsilon'(\nu) + j\epsilon''(\nu), \tag{S2-1}$$

where ν is frequency (Hz), $\epsilon'(\nu)$ is the real part (dimensionless), and $\epsilon''(\nu)$ is the imaginary part (dimensionless).

The complex refractive index is:

$$\tilde{n}(\nu) = n(\nu) + jk(\nu), \quad \tilde{n}^2(\nu) = \epsilon_r(\nu), \tag{S2-2}$$

where $n(\nu)$ is the refractive index (dimensionless), and $k(\nu)$ is the extinction coefficient (dimensionless).

Separating real and imaginary parts:

$$n^2(\nu) - k^2(\nu) = \epsilon'(\nu), \tag{S2-3}$$

$$2n(\nu)k(\nu) = \epsilon''(\nu). \tag{S2-4}$$

Solving:

$$n(\nu) = \sqrt{\frac{1}{2}\left(\epsilon'(\nu) + \sqrt{\epsilon'^2(\nu) + \epsilon''^2(\nu)}\right)}, \tag{S2-5}$$

$$k(\nu) = \sqrt{\frac{1}{2}\left(-\epsilon'(\nu) + \sqrt{\epsilon'^2(\nu) + \epsilon''^2(\nu)}\right)}. \tag{S2-6}$$

The absorption coefficient is given by:

$$\alpha(\nu) = \frac{4\pi\nu}{c}\, k(\nu), \tag{S2-7}$$

where $\alpha(\nu)$ is the absorption coefficient (m^{-1}), and c is the speed of light (m/s). Other constitutive relations between dielectric susceptibility, polarization and permittivity, used in further derivations, are the following:

$$\mathbf{P}(\nu) = \varepsilon_0\, \chi(\nu)\, \mathbf{E}(\nu), \tag{S2-8}$$

$$\varepsilon(\nu) = \varepsilon_0[1 + \chi(\nu)] \equiv \varepsilon_0\, \varepsilon_r(\nu), \tag{S2-9}$$

$$\chi(\nu) = \frac{\varepsilon(\nu)}{\varepsilon_0} - 1, \tag{S2-10}$$

$$\varepsilon'(\nu) = \varepsilon_0\big[1 + \chi'(\nu)\big], \qquad \varepsilon''(\nu) = \varepsilon_0\, \chi''(\nu). \tag{S2-11}$$

with ϵ_0: vacuum permittivity (F/m = $C^2 \cdot s^2/(kg \cdot m^3)$).

S2-3 From the Lorentz Oscillator to Permittivity and Absorption for Single Mode

For a protein, as for any other system carrying a complex electric charge distribution, the motion (i.e. system deformation) due to each vibrational mode l is associated with a change of the dipole moment. For each mode, stemming from the equation of motion for a damped harmonic oscillator (oscillating mass point carrying a charge) driven by an external electric field [1], this situation can be represented as a particle with effective (modal) mass m_l and effective charge q_l associated with the change of the dipole moment p_l [2]:

$$\ddot{p}_l + 2\pi\gamma_l\dot{p}_l + (2\pi\nu_l)^2 p_l = \frac{\rho_l}{m_l}E(t), \tag{S2-12}$$

where m_l: mass of the particle (or modal) mass (kg), γ_l: damping constant (Hz), ν_l: resonance frequency (Hz), p_l: dipole moment (C.m) related to particular mode induced by external field E (C.m), ρ_l: effective charge (also called as dipole derivative, see lower section for more details) (C), E: electric field strength (V/m). Here we assume, for simplicity, that the E and x are along the same vector direction.

Assuming harmonically time varying external electric field $E(t) = E_0 \mathrm{e}^{-j2\pi\nu t}$ the steady-state solution in frequency domain:

$$p_l(\nu) = \frac{\rho_l}{m_l}\frac{E(\nu)}{4\pi^2\left[\nu_l^2 - \nu^2 - j\nu\gamma_l\right]} \tag{S2-13}$$

which is the dipole moment (units of (C·m)) induced by the external electric field $E(\nu)$.

The macroscopic polarization due to randomly oriented dipoles is written as:

$$P(\nu) = \frac{1}{3}N_V\, p_l(\nu), \tag{S2-14}$$

where N_V is number volumetric density of molecules ($/\mathrm{m}^3$), P is polarization ($\mathrm{C/m^2}$), and the factor $\frac{1}{3}$ is due to random orientation, see supplementary info from [3] for derivation.

Relative dielectric susceptibility contribution due to a single vibrational mode l is obtained by combining equations S2-8, S2-13, S2-14:

$$\chi_l(\nu) = \frac{P(\nu)}{E(\nu)\epsilon_0} = \frac{\rho_l N_V}{\epsilon_0 m_l}\frac{1}{4\pi^2\left[\nu_l^2 - \nu^2 - j\nu\gamma_l\right]} \tag{S2-15}$$

S2-4 Real and Imaginary Part of Permittivity and Absorption

We use complex conjugation identities:

$$\frac{1}{A - jB} = \frac{A + jB}{A^2 + B^2}$$

Let:

$$A = \nu_l^2 - \nu^2, \quad B = \nu\gamma_l$$

Then:

$$\chi'(\nu) = \frac{N_V \rho_l^2}{\epsilon_0 m_l} \cdot \frac{A}{A^2 + B^2} = \frac{N_V \rho_l^2}{\epsilon_0 m_l} \cdot \frac{\nu_l^2 - \nu^2}{(\nu_l^2 - \nu^2)^2 + (\nu\gamma_l)^2}, \tag{S2-16}$$

$$\chi''(\nu) = \frac{N_V \rho_l^2}{\epsilon_0 m_l} \cdot \frac{B}{A^2 + B^2} = \frac{N_V \rho_l^2}{\epsilon_0 m_l} \cdot \frac{\nu\gamma_l}{(\nu_l^2 - \nu^2)^2 + (\nu\gamma_l)^2}. \tag{S2-17}$$

Considering Eqs.S2-5 and S2-7 and

$$\epsilon'(\nu) = \epsilon_0 \epsilon_r'(\nu) = \epsilon_0(1 + \chi'(\nu)) \tag{S2-18}$$

$$\epsilon''(\nu) = \epsilon_0 \epsilon_r''(\nu) = \epsilon_0 \chi''(\nu) \tag{S2-19}$$

then the absorption coefficient is:

$$\alpha(\nu) = \frac{4\pi\nu}{c} \cdot \sqrt{\frac{1}{2}\left(-\epsilon'(\nu) + \sqrt{\epsilon'^2(\nu) + \epsilon''^2(\nu)}\right)}. \tag{S2-20}$$

S2-5 Effective Charge (Dipole Variation) and How to Obtain it from Modal Eigenvectors and Partial Charges of Atoms

GROMACS Normal Mode Analysis (*nmeig* function) of a protein structure outputs eigenvector and frequency of each mode. The frequency ν_l is output in the units of cm^{-1} (wavenumbers), hence needs to be recalculated to appropriate units.

The eigenvector $\mathbf{e}_{l,i\alpha}$ of each mode l represents the relative displacement of each atom i (α represents coordinate system components, e.g. x,y,z) within the mode, therefore are dimensionless. To implement the eigenvector from GROMACS Normal Mode Analysis, one needs to understand its effective units. GROMACS outputs eigenvectors so that they have effective units of $1/\sqrt{m}$, see Appendix S2-9 for explanation. Effective charge (a.k.a variation of dipole moment, dipole variation, dipole derivative) for each particle (grain, in our case of all atom simulation, it is each atomic group) is calculated as

$$^{m}\boldsymbol{\rho}_{li} = \mathbf{e}_{l,i} q_i \tag{S2-21}$$

where q_i is the charge of the i-th atom. Note that the effective charge $^{m}\boldsymbol{\rho}_{l,i}$ is a vector. We use left upper index m to denote that $^{m}\boldsymbol{\rho}_{l,i}$ has units of $\frac{charge}{\sqrt{mass}}$. The effective charge for the mode l is the vectorial sum of the effective charges of all atomic groups for the particular mode l:

$$^{m}\boldsymbol{\rho}_l = \sum_i^N q_i \mathbf{e}_{li} = (\sum_i^N q_i e_{lix})\mathbf{x}_0 + (\sum_i^N q_i e_{liy})\mathbf{y}_0 + (\sum_i^N q_i e_{liz})\mathbf{z}_0 \tag{S2-22}$$

where $\mathbf{x}_0, \mathbf{y}_0, \mathbf{z}_0$ are unit vectors in Cartesian coordinate system. Then, the magnitude of ρ_l is

$$^{m}\rho_l = \|^{m}\boldsymbol{\rho}_l\| = \sqrt{(\sum_i^N q_i e_{lix})^2 + (\sum_i^N q_i e_{liy})^2 + (\sum_i^N q_i e_{liz})^2} \tag{S2-23}$$

Note that in Gromacs, the charges have units of elementary charges and mass is in dalton so the appropriate factors needs to used when calculating the absolute values in SI or other appropriate units.

Using the ${}^{m}\rho_l$ which encompasses the mass involved in the mode l, the Eq.S2-15 can be written as:

$$\chi_l(\nu) = \frac{N_V}{\epsilon_0 4\pi^2} \frac{{}^{m}\rho_l^2}{(\nu_l^2 - \nu^2 - j\nu\gamma_l)} \tag{S2-24}$$

S2-6 Generalization to Multiple Modes

For M modes (effective oscillators) per molecule, the susceptibility is a sum of contributions of individual modes:

$$\chi(\nu) = \frac{N_V}{\epsilon_0 4\pi^2} \sum_{l}^{M} \frac{{}^{m}\rho_l^2}{(\nu_l^2 - \nu^2 - j\nu\gamma_l)} \tag{S2-25}$$

This equation assumes that all modes have their ${}^{m}\boldsymbol{\rho}_l$ vectors aligned with the external electric field so that they add to the susceptibility with the same weight. For experimental spectroscopic predictions, we assume that we probe a large (high number of molecules) but dilute (so that the molecules and their electromagnetic response don't interact) ensemble of molecules which are randomly angularly oriented with respect to electric field vector of the external EM field. To account for the random angular distribution of molecules (hence randomly oriented vectors of effective charge) with respect to external electric field vector (assuming linear polarization), one has to add a prefactor of 1/3. This prefactor comes from a solution of integral addressing this issue, see Supplementary information in [3] for derivation. Hence the susceptibility from Eq. S2-25 becomes

$$\chi(\nu) = \frac{1}{3} \frac{N_V}{\epsilon_0 4\pi^2} \sum_{l}^{M} \frac{{}^{m}\rho_l^2}{(\nu_l^2 - \nu^2 - j\nu\gamma_l)} \tag{S2-26}$$

Real and imaginary parts:

$$\chi'(\nu) = \frac{N_V}{\epsilon_0 12\pi^2} \sum_{l}^{M} \frac{{}^{m}\rho_l^2 A_l}{A_l^2 + B_l^2}, \tag{S2-27}$$

$$\chi''(\nu) = \frac{N_V}{\epsilon_0 12\pi^2} \sum_{l}^{M} \frac{{}^{m}\rho_l^2 B_l}{A_l^2 + B_l^2}, \tag{S2-28}$$

with $A = \nu_l^2 - \nu^2, \quad B = \nu\gamma_l$.

Absorption:

$$\alpha(\nu) = \frac{4\pi\nu}{c} \cdot \sqrt{\frac{1}{2}\left(-\epsilon'(\nu) + \sqrt{\epsilon'^2(\nu) + \epsilon''^2(\nu)}\right)}. \tag{S2-29}$$

S2-7 Conversion of Chemical Units to Number Density

To convert a concentration from common chemical units such as mg/mL or mol/L to the number of molecules per cubic meter (denoted by N_V), we follow a systematic approach. For a concentration given in mg/mL, note that

$$1\,\text{mg/mL} = 1\,\text{g/L},$$

since 1 g = 1000 mg and there are 1000 mL in 1 L. If the substance has a molecular weight M_w (in g/mol), the concentration in g/L can be converted to mol/L by dividing by M_w:

$$\text{Concentration in mol/L} = \frac{\text{Concentration in g/L}}{M_w}.$$

Because $1\,\text{L} = 10^{-3}\,\text{m}^3$, multiplying the molar concentration by 10^3 converts it to mol/m^3:

$$\text{Concentration in mol/m}^3 = \frac{\text{Concentration in g/L}}{M_w} \times 10^3.$$

Finally, multiplying by Avogadro's number, $N_A \approx 6.022 \times 10^{23}$ molecules/mol, yields the number of molecules per cubic meter:

$$N_V = \frac{\text{Concentration in g/L}}{M_w} \times 10^3 \times N_A.$$

For a concentration already expressed in mol/L, the conversion is even more straightforward. Multiply by 10^3 to convert to mol/m^3 and then by N_A to obtain:

$$N_V = \text{Concentration in mol/L} \times 10^3 \times N_A.$$

S2-8 Limitations of the NMA Approach for Calculating Absorption

- Assumes harmonic potential; real systems may be anharmonic.
- Assumes linear response to field; nonlinear effects ignored.
- No coupling between modes included.
- Damping is phenomenological.
- Thermal fluctuations do not enter driven permittivity response.

S2-9 Appendix: Clarification on GROMACS NMA eigenvectors: notation, properties, and units

Let $H \in \mathbb{R}^{3N\times 3N}$ be the Cartesian Hessian and $M = \text{diag}(m_1 I_3, \ldots, m_N I_3)$ the mass matrix. The mass-weighted Hessian is

$$\tilde{H} \;=\; M^{-1/2}\, H\, M^{-1/2}. \tag{S2-30}$$

GROMACS `gmx nmeig` diagonalizes $\tilde{H}$ to obtain the mass-basis (mass-weighted) eigenvectors $\mathbf{e}_l^{(m)}$ and eigenvalues λ_l:

$$\tilde{H}\,\mathbf{e}_l^{(m)} \;=\; \lambda_l\,\mathbf{e}_l^{(m)}, \qquad \big(\mathbf{e}_l^{(m)}\big)^{\top}\mathbf{e}_k^{(m)} = \delta_{lk}. \tag{S2-31}$$

Before writing output, GROMACS scales them back to Cartesian coordinates:

$$\mathbf{e}_l \;=\; M^{-1/2}\,\mathbf{e}_l^{(m)}, \qquad \text{equivalently} \qquad \mathbf{e}_l^{(m)} \;=\; M^{1/2}\,\mathbf{e}_l. \tag{S2-32}$$

Component-wise, for atom i and Cartesian component $\alpha \in \{x, y, z\}$,

$$e_{l,i\alpha} \;=\; \frac{e_{l,i\alpha}^{(m)}}{\sqrt{m_i}}. \tag{S2-33}$$

S2-9.1 What GROMACS outputs (`gmx nmeig`).

1. The final eigenvectors written by `gmx nmeig` are the Cartesian vectors $\mathbf{e}_l$ that carry a per-atom factor $1/\sqrt{m_i}$ [Eq. (S2-32)–(S2-33)]. Using the GROMACS option `-m` (default: yes) divides Hessian elements by the product of $\sqrt{m}$ of the involved atoms prior to diagonalization (recommended for "Normal Modes" analysis; see `gmx nmeig` manual).

2. Indexing: we denote the Cartesian output as $e_{l,i\alpha}$ where l is the mode index, i the atom index, and $\alpha \in \{x, y, z\}$.

S2-9.2 Orthogonality and normalization.

1. The Cartesian eigenvectors $\mathbf{e}_l$ are generally *not* orthonormal under the standard Euclidean dot product: $\mathbf{e}_l^\top \mathbf{e}_k \neq \delta_{lk}$.

2. They *are* orthonormal in the mass-weighted inner product:

$$\mathbf{e}_l^\top M \mathbf{e}_k \;=\; \delta_{lk}. \tag{S2-34}$$

3. The mass-basis vectors $\mathbf{e}_l^{(m)}$ are Euclidean-orthonormal and unit-norm by construction [Eq. (S2-31)].

4. This matches the GROMACS manual note: when mass-weighting is used, eigenvectors are scaled back to Cartesian coordinates for output; thus they are exactly orthogonal only in the mass-weighted norm (not in the plain Cartesian norm).

S2-9.3 Units / dimensions.

1. Mass-basis vectors $\mathbf{e}_l^{(m)}$ are dimensionless basis vectors (unit norm, orthogonal).

2. Cartesian output vectors $\mathbf{e}_l$ have per-component dimensions $[\, e_{l,i\alpha} \,] = \text{mass}^{-1/2}$ (i.e., $1/\sqrt{\text{mass}}$); "Cartesian" here refers to the x, y, z components, not to having units of length.

3. For our permittivity calculations we therefore use the Cartesian $\mathbf{e}_l$, which explicitly introduce the required $1/\sqrt{m_i}$ factors and yield the correct physical dimensions.

S2-9.4 Practical note on GROMACS option.

The manual entry for `gmx nmeig` states (paraphrased): `-[no]m (yes)` divides Hessian elements by the product of $\sqrt{\text{mass}}$ of the involved atoms before diagonalization; this should be used for Normal Modes analysis (see the online help for `gmx nmeig`).

S2-10 Summary of Units and Dimensions

Unless stated otherwise, SI units are used. The mode index is l; the atom index is i.

- **Frequencies and rates:**
  ν, ν_l — frequency (Hz).
  $\omega = 2\pi\nu$ — angular frequency ($\text{rad}\,\text{s}^{-1}$).
  γ_l — damping rate (s^{-1}).

- **Fields and material response:**
  E — electric field (V/m).
  P — polarization (C/m^2).
  $\varepsilon(\nu)$ — absolute permittivity (F/m).
  $\varepsilon_0 = 8.854\,187\,817 \times 10^{-12}\,\mathrm{F/m}$ (also $\mathrm{C^2 s^2/(kg\,m^3)}$).
  $\varepsilon_r(\nu) = \varepsilon(\nu)/\varepsilon_0$ — relative permittivity (dimensionless).
  $\chi(\nu)$ — electric susceptibility (dimensionless).

- **Optical constants and absorption:**
  $n(\nu)$ — refractive index (dimensionless).
  $k(\nu)$ — extinction coefficient (dimensionless).
  $\alpha(\nu)$ — absorption coefficient (m^{-1}).
  $c = 2.997\,924\,58 \times 10^8\,\mathrm{m/s}$ — speed of light.

- **Microscopic oscillator (mode) quantities:**
  m_l — effective (modal) mass (kg).
  x_l — modal displacement (m).
  p_l — induced dipole moment of mode l (C·m).

- **Charges (atomic and effective):**
  q_i — partial charge of atom i (C); in GROMACS topologies typically in units of e with $1e = 1.602\,176\,634 \times 10^{-19}\,\mathrm{C}$.
  ρ_l — unweighted effective charge ("dipole derivative") used with m_l (C).
  ${}^m\boldsymbol{\rho}_l = \sum_i q_i\, \mathbf{e}_{l,i}$ — mass–weighted effective charge vector (units $\mathrm{C\,kg^{-1/2}}$).
  ${}^m\rho_l = \|{}^m\boldsymbol{\rho}_l\|$ — magnitude of mass–weighted effective charge ($\mathrm{C\,kg^{-1/2}}$).

- **Eigenvectors (NMA):**
  $\mathbf{e}^{(m)}_{l,i\alpha}$ — mass–basis eigenvector (dimensionless).
  $\mathbf{e}_{l,i\alpha}$ — GROMACS Cartesian output with units mass$^{-1/2}$ (kg$^{-1/2}$); $\alpha \in \{x, y, z\}$.

- **Lorentz helpers**
  $A_l(\nu) = \nu_l^2 - \nu^2$ (s^{-2}).
  $B_l(\nu) = \nu\,\gamma_l$ (s^{-2}).

- **Number densities and constants:**
  N_V — number (molecular) density (m^{-3}).
  $N_A = 6.022\,140\,76 \times 10^{23}\,\mathrm{mol^{-1}}$ — Avogadro constant.
  M_w — molecular weight (g/mol); convert to kg/mol via $10^{-3} M_w$.

- **Orientation averaging:**
  For a dilute ensemble of randomly oriented dipoles and a linearly polarized probe field, the orientational average contributes a factor 1/3 (dimensionless) to χ.

- **Wavenumber–frequency conversion (spectroscopy):**
  If a mode frequency is given as wavenumber $\tilde{\nu}$ (cm^{-1}), then $\nu\,[\mathrm{Hz}] = (2.997\,924\,58 \times 10^{10})\,\tilde{\nu}\,[\mathrm{cm^{-1}}]$.

S2-11 References

References

[1] Wooten, F. *Optical properties of solids* (Academic Press, 1972).

[2] Riffe, D. M. Canonical Models of Dielectric Response. *arXiv:1806.05158 [physics]* (2018). ArXiv: 1806.05158.

[3] Krivosudský, O. & Cifra, M. Microwave absorption by nanoresonator vibrations tuned with surface modification. *EPL (Europhysics Letters)* **115**, 44003 (2016).
